# Identifying motor learning deficits in neurological conditions: a critical analysis of a perennial problem

**DOI:** 10.1007/s00221-026-07237-6

**Published:** 2026-02-12

**Authors:** Rajiv Ranganathan, Sharo Costa, Angelyn May Mengote, Megan Thomas, Rohan Ved

**Affiliations:** 1 Department of Kinesiology, Michigan State University, 308 W Circle Dr, MI East Lansing, USA; 2 Department of Mechanical Engineering, Michigan State University, East Lansing, MI USA; 3 College of Osteopathic Medicine, Michigan State University, East Lansing, MI USA

**Keywords:** Baseline, Skill, Learning, Performance, Parkinson’s disease, Stroke

## Abstract

Identifying deficits in motor learning has the potential to serve as an indicator of brain function in several neurological conditions. However, evidence for motor learning deficits can be confounded by factors unrelated to learning. In this review, we critically examine the evidence for these deficits in neurological conditions, focusing both on conceptual and methodological issues. Across a wide range of neurological conditions, we found that a majority of evidence for motor learning deficits is difficult to clearly interpret as learning-related and is potentially confounded by the presence of baseline differences. In addition, the use of low sample sizes, short time durations of practice, and the narrow focus on sequence learning paradigms also raise questions about the validity and generality of this evidence. Given that deficits in motor learning have implications for the early detection of neurological status and the design of rehabilitation strategies, we highlight the need for greater rigor when addressing this important, but perennially challenging, question.

## Introduction

There has been a long-standing interest in understanding motor learning differences associated with neurological conditions (Milner 1965; Corkin 1968; Heilman et al. 1975; Sanes et al. 1990). Although some motor learning studies have focused on conditioning of responses and reflexes (Glickstein and Yeo 1990; Thompson et al. 2009), here we focus on motor learning in the context of practice-related improvements in task performance. This process of motor learning has been associated with several different brain regions, including the basal ganglia, cerebellum, primary motor cortex, and premotor cortex, and depends on a number of factors such as the type of task being learned and the stage of learning (Willingham 1998; Doyon and Benali 2005; Seidler 2010; Penhune and Steele 2012; Spampinato and Celnik 2021). In light of these findings, identifying deficits in motor learning has been viewed as having the potential to reveal unique insights into neurological function, which have implications for early non-invasive identification of disease states, and for designing effective rehabilitation strategies.

Deficits in motor learning have been reported in a wide range of neurological conditions including Parkinson’s disease (Harrington et al. 1990; Krebs et al. 2001), stroke (Boyd and Winstein 2004), Alzheimer’s disease (Ferraro et al. 1993; Rouleau et al. 2002), autism (Mostofsky et al. 2000) and dyslexia (Stoodley and Stein 2006). However, the evidence for such learning differences has been somewhat mixed as several studies show that motor learning may not be affected in these populations (Grafman et al. 1990; Agostino et al. 1996; Winstein et al. 1999). There have been attempts to resolve these conflicting findings by distinguishing between the type of task, level of impairment, extremity tested, or the learning mechanism involved (Ashe et al. 2006; van Halteren-van Tilborg et al. 2007; Nieuwboer et al. 2009; Marinelli et al. 2017). However, given that motor learning encompasses several processes (Krakauer et al. 2019), these mixed findings raise the need for a greater understanding of both conceptual and methodological aspects of how such deficits are quantified across studies (Ranganathan et al. 2022c).

**Fig. 1:**
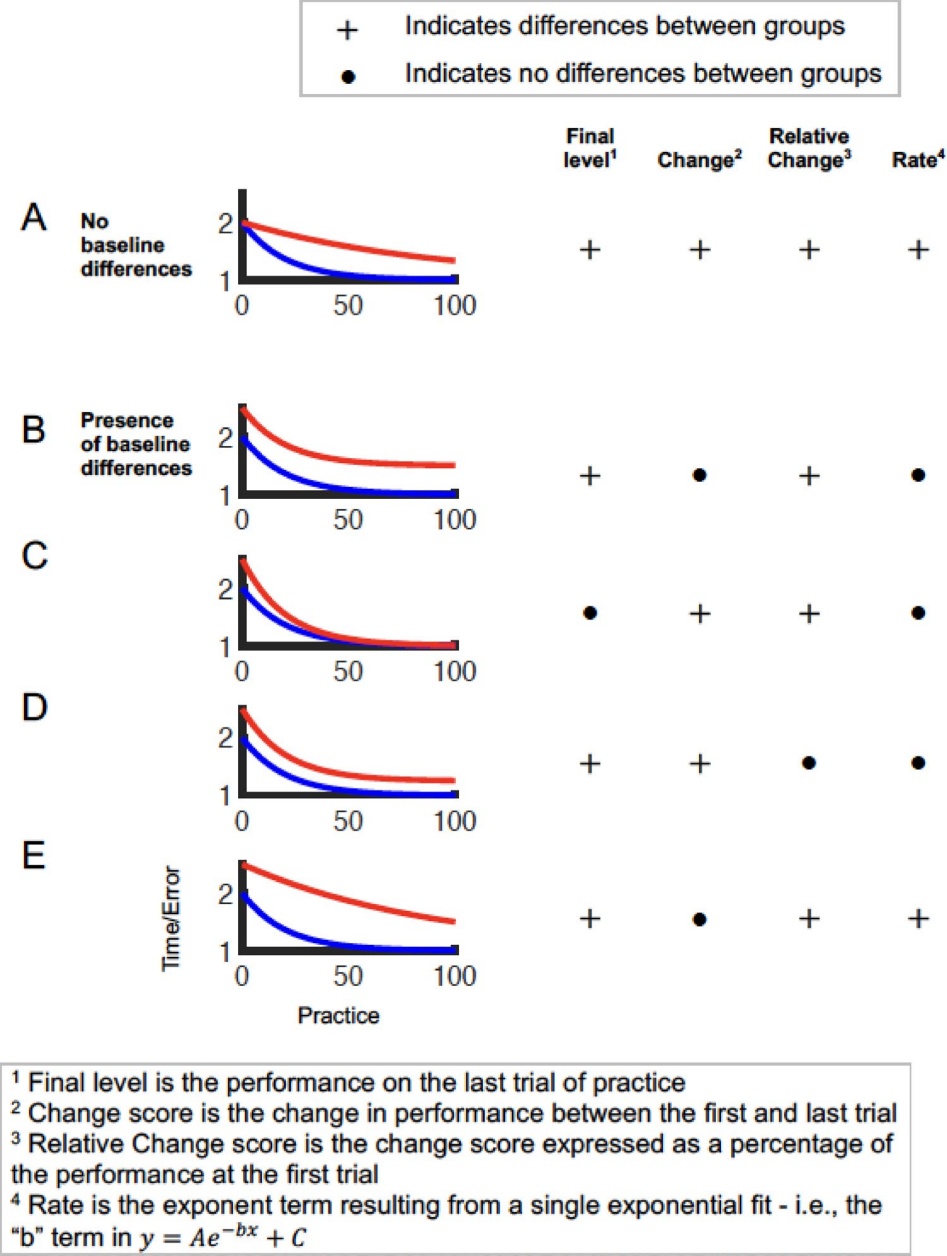
Assessing learning deficits in the presence of baseline differences. Hypothetical learning curves are presented for two groups (indicated in blue and red), with practice on the x-axis and movement time/error on the y-axis in arbitrary units (such that decreasing values indicate “better” performance). Learning deficits are assessed using four different learning measures (final level, change, relative change, and rate). When there are no baseline differences, **A** all measures of learning tend to agree. However, in the presence of baseline differences (**B**)–(**E**), conclusions can be ambiguous because they are highly dependent on the choice of the learning measure

Addressing the question of whether there is a motor learning deficit has several challenges, primarily because of the “learning-performance distinction” (Salmoni et al. 1984; Schmidt and Bjork 1992; Kantak and Winstein 2012). Because motor learning is inferred based on observed behavior, several factors (such as fatigue, motivation, or attention) can influence performance without affecting learning. Specifically, in the context of neurological conditions, one factor that complicates this issue is that clinical populations often do not start at the same initial level of motor performance as age-matched unimpaired controls (i.e., they are at a different ‘baseline’ prior to learning). This means that the same results can be interpreted in different (sometimes even contradictory) ways depending on how learning is defined (Schmidt 1972; Kitago and Krakauer 2010). For example, when there are no group differences at baseline, different measures of learning using absolute measures (such as final performance level) or relative measures (such as change or relative change scores) will tend to result in the same conclusion (Fig. 1A). However, when the baseline levels are different, the question of which group “learned more” depends on how learning is measured. For example, consider two groups that have the same exponential learning rate, but plateau at different levels (Fig. 1B). In this case, using measures such as final performance level or relative change could indicate differences between groups, whereas using measures a learning rate or a change score could indicate no differences between the groups (depending on the exact values of baseline and plateau performance levels). Similarly, other such inconsistencies among learning metrics in the presence of baseline differences can be shown where the two groups have (i) same learning rates but different change scores (Fig. 1C), (ii) same learning rates but different relative change scores (Fig. 1D), and (iii) different learning rates but same change scores (Fig. 1E). These examples highlight the conceptual difficulty in measuring learning when baseline levels are different between groups. In addition to these conceptual factors, methodological factors associated with the evidence for learning deficits also need to be considered because they determine the strength of the evidence for a deficit (e.g., the sample size), as well as the implications of these findings for real-world applications such as rehabilitation (e.g., the type of task and the time duration of practice).

To characterize the prevalence of these issues in the literature, we reviewed studies that address the issue of motor learning deficits in neurological populations. Given the wide range of experimental paradigms used, we use the term ‘learning’ here to include any practice-dependent changes in task performance (without making the distinction between transient and persistent changes as highlighted in the learning-performance distinction (Salmoni et al. 1984; Schmidt and Bjork 1992; Kantak and Winstein 2012)). Moreover, because our focus was on the measurement of motor learning deficits in general, we sampled across a broad set of neurological conditions and critically evaluate the evidence provided for motor learning differences. We highlight critical conceptual and methodological issues involved in such studies and provide guidelines for future studies to address these questions with greater rigor.

## Methods

The Web of Science database was used for the literature search, with the following keywords: “motor learning deficits”, “stroke and motor learning”, “motor learning and rehabilitation”, and “motor learning and neurological deficits”. Papers generated from the search (*n* = 268) were screened based on the following inclusion criteria: (i) the paper was an experimental paper, (ii) it was based on human-subject studies with a focus on neurological conditions, (iii) there was the presence of a control group for comparison, and (iv) the study tested a motor learning component (i.e., participants practiced a motor task over multiple trials with performance tracked over time/trials). It is important to note that because the focus of our review was to examine practices over a wide range of motor learning studies, we did not restrict our review based on any specific definitions (theoretical or practical) or measurements of motor learning.

**Fig. 2:**
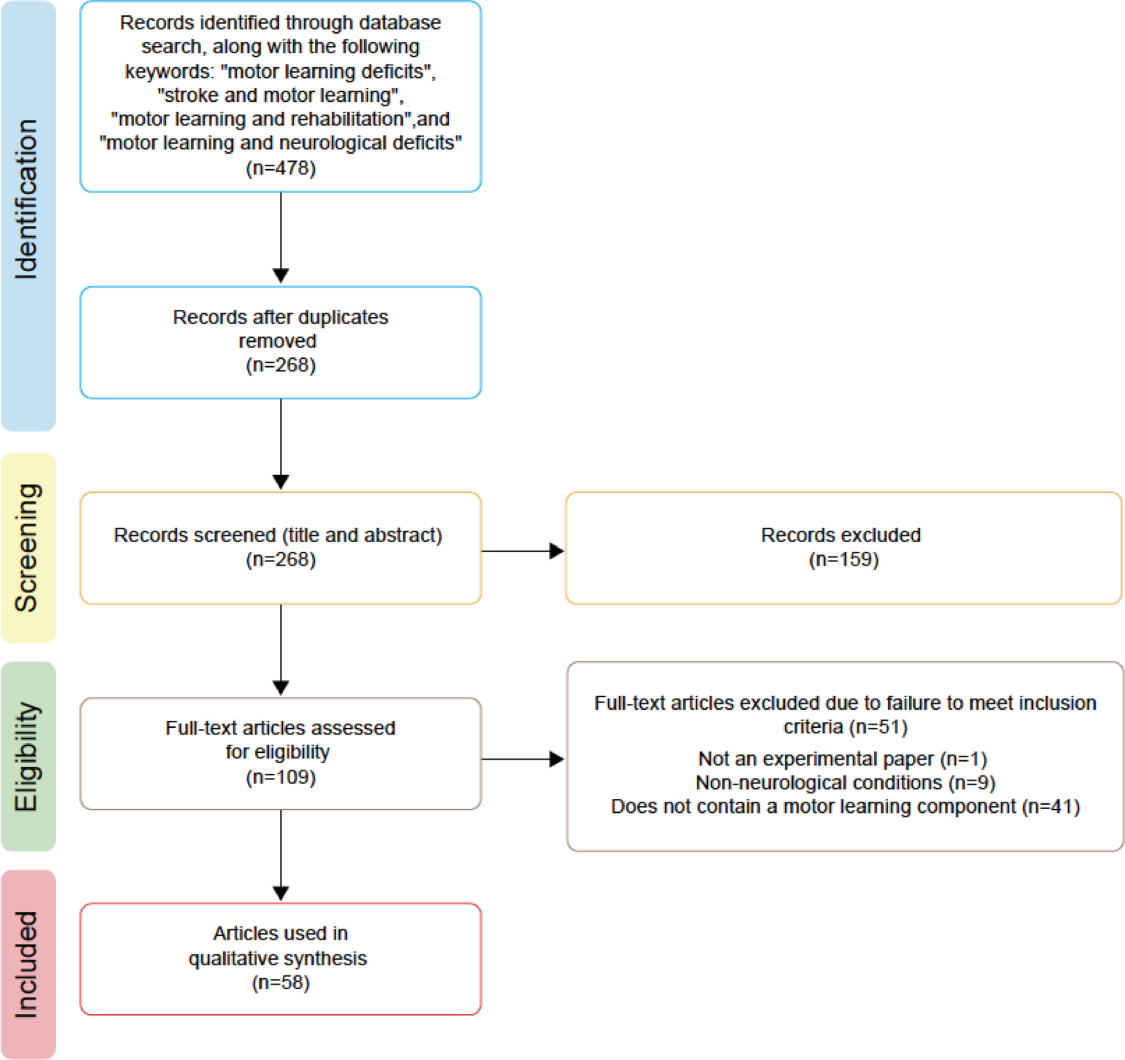
PRISMA graph of study selection. A total of 58 papers (covering 72 studies) on motor learning deficits were included for this review

All studies that did not meet any one of the inclusion criteria listed above, were excluded. The studies were screened twice, each time by a different author, to make sure they met the study criteria, and a third author acted as a tie-breaker when needed. For papers reporting multiple experiments or multiple tasks, each experiment was considered a separate study. Overall, a total of 58 articles (covering 72 studies) were included based on the criteria that were then subject to a full-text review (Fig. 2).

## Data analysis

For each included study, the following data were extracted: (i) the neurological condition being investigated, (ii) the type of task studied, (iii) whether the study made a claim of a learning deficit, (iv) the type of deficit being examined, (v) the type of learning measure being examined, (vi) whether there were initial baseline differences in motor performance between experimental and control groups, and (vii) strategies to account for baseline differences, (viii) average group sample size, and (ix) the time duration of motor learning.

The neurological condition being investigated was based on the description of the participant group. The type of task was selected based on the classification from (Ranganathan et al. 2021), which divides the task into one of six categories - adaptation, applied, coordination, sequence learning, tracking, and variability. Briefly, adaptation tasks involved modification of well-learned movements (e.g., visuomotor rotation), applied tasks involved real-world tasks (e.g., a soccer kick), coordination tasks involved producing a spatiotemporal pattern using more than a single degree of freedom (e.g., bimanual coordination), sequence learning tasks involved producing a sequence of several movement responses (e.g., key pressing), tracking tasks involved producing a time-varying spatiotemporal pattern (e.g., tracking a moving target), and variability tasks involved producing a steady-state performance (e.g., throwing to a specific target). As with prior work, although some tasks did not fall exclusively into one category, we decided to code each task into the ‘best fitting’ category. The rationale behind coding the type of task was to examine the underlying learning mechanisms involved in learning.

The claim of a learning deficit was coded as a “yes”, “no”, or “unclear” based on the authors’ interpretation of the results. The type of deficit being examined was classified either as a “learning mechanism” or a “rate” deficit. Learning mechanism deficits involved comparison of learning in two different conditions (e.g., as characterized by a group × condition interaction). Rate deficits involved comparison of how ‘quickly’ participants learned the task relative to controls (e.g., characterized by a group × time interaction or a learning rate parameter comparison). The type of learning measure used to infer the deficit was classified based on one of four categories – (i) absolute level: the value of the dependent variable is directly compared between groups, (ii) change score: the difference in the dependent variable (between two or more conditions or time points) is compared between groups, (iii) normalized change: the ‘normalized’ or ‘relative’ difference (e.g., a % improvement) in the dependent variable (between two or more conditions or time points) is compared between groups and (iv) learning rate: the learning rate (e.g., as estimated by linear slopes or exponential parameters) is compared between groups.

Baseline difference was coded as a “yes” (baseline difference in performance between the experimental and control groups was reported in the manuscript and this difference was statistically significant), “no” (baseline difference in performance between the experimental and control groups was reported in the manuscript and this difference was not statistically significant) or “unclear” (no statistical comparisons were reported, or statistical comparisons were not made between the groups under identical task conditions).

Strategies to account for baseline differences were coded as: (i) using within-subject control - i.e., comparing the participant’s performance relative to their own performance in another condition, (ii) statistical adjustments (e.g., adding covariates or normalizing the dependent variable), (iii) task modification – i.e., modifying the task to make performance equivalent between groups (e.g., changing the target speed in a tracking task), or (iv) none.

The sample size of the study was determined as the average number of participants per group in the experiment (i.e., total sample size divided by number of groups; so this number could be a non-integer). Time duration was coded based on the length of time (measured in days) of practice, as a measure of its relevance toward capturing long-term learning and rehabilitation.

## Results

The full table of articles with references is available at https://osf.io/v6af4/. The main results are summarized below.

### Motor learning deficits were reported across several neurological conditions

**Fig. 3:**
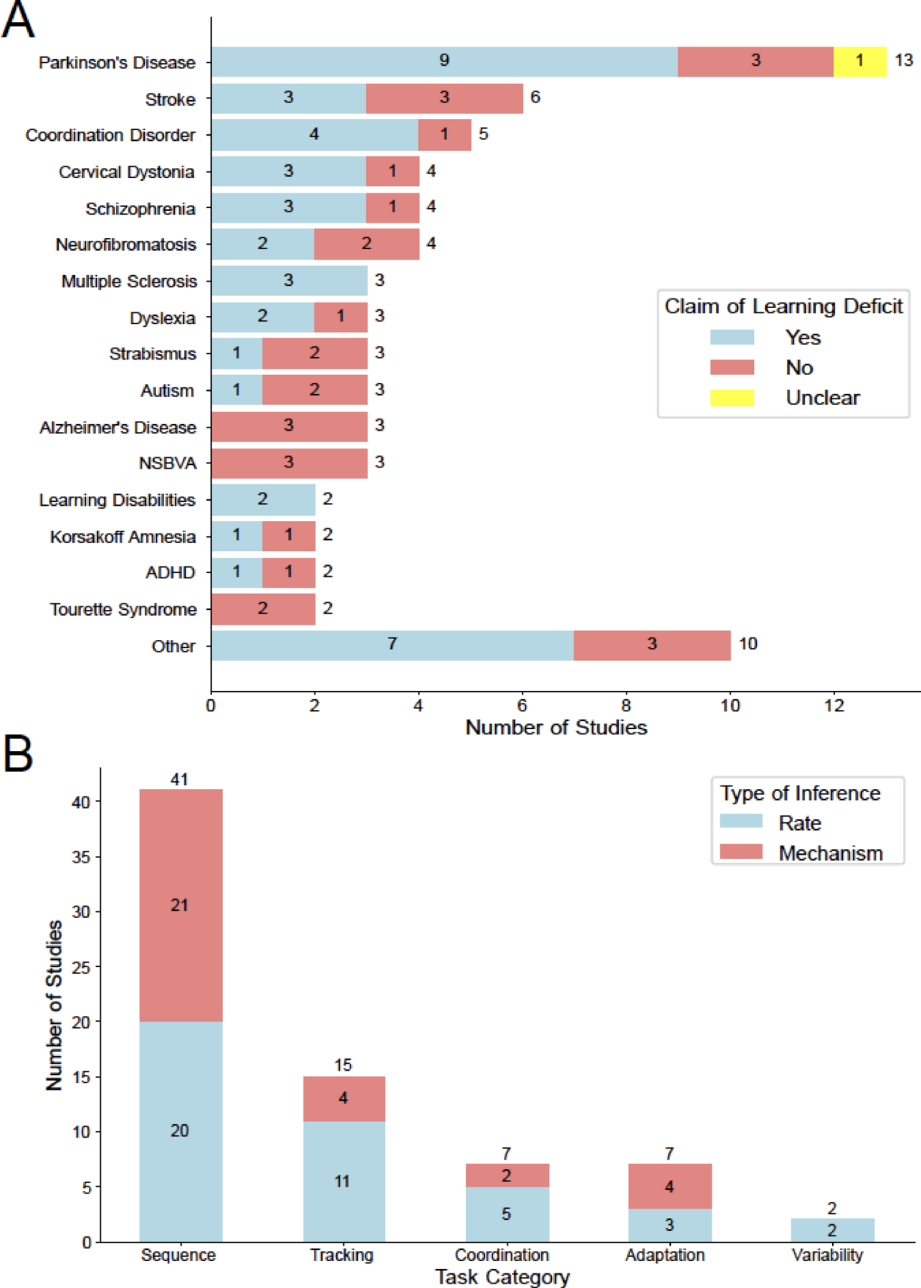
**A** Neurological conditions where motor learning deficits were assessed. A majority of the studies (57%) reported deficits in motor learning. **B** Types of task paradigms used for assessing deficits and the nature of the deficit investigated. Sequence learning was the most popular paradigm, followed by adaptation and coordination tasks. Learning rate deficits were investigated more often, although sequence learning paradigms did tend to have a high number of studies that investigated learning mechanisms

The review included several neurological conditions (Fig. 3A), with the top categories being Parkinson’s disease (*n* = 13, 26.4%), stroke (*n* = 6, 8.3%), and developmental coordination disorder (*n* = 5, 6.9%). We found that the presence of motor learning deficits was reported in a majority of these studies (*n* = 42, 58.3%).

### Sequence learning was the dominant task paradigm, and the types of learning deficits examined were influenced by the task paradigm

The analysis of task category showed that sequence learning tasks were the dominant paradigm, used in over twice as many studies (*n* = 41, 56.9%) as the next highest task category (Fig. 3B). Tracking (*n* = 15, 20.8%) was the next most common category, followed by coordination (*n* = 7, 9.7%), adaptation (*n* = 7, 9.7%), and variability (*n* = 2, 2.8%) tasks.

The analysis of the type of motor learning deficits predominantly depended on the type of task paradigm (Fig. 3B). Studies examining deficits in learning rates were more common overall (*n* = 41, 56.9%). However, sequence learning (*n* = 21 out of 41, 51.2%) and adaptation paradigms (*n* = 4 out of 7, 57%) tended to have a fairly good representation of studies examining deficits in learning mechanisms.

### Baseline differences in motor performance were reported frequently, and strategies to account for baseline differences depended on the type of task paradigm

**Fig. 4:**
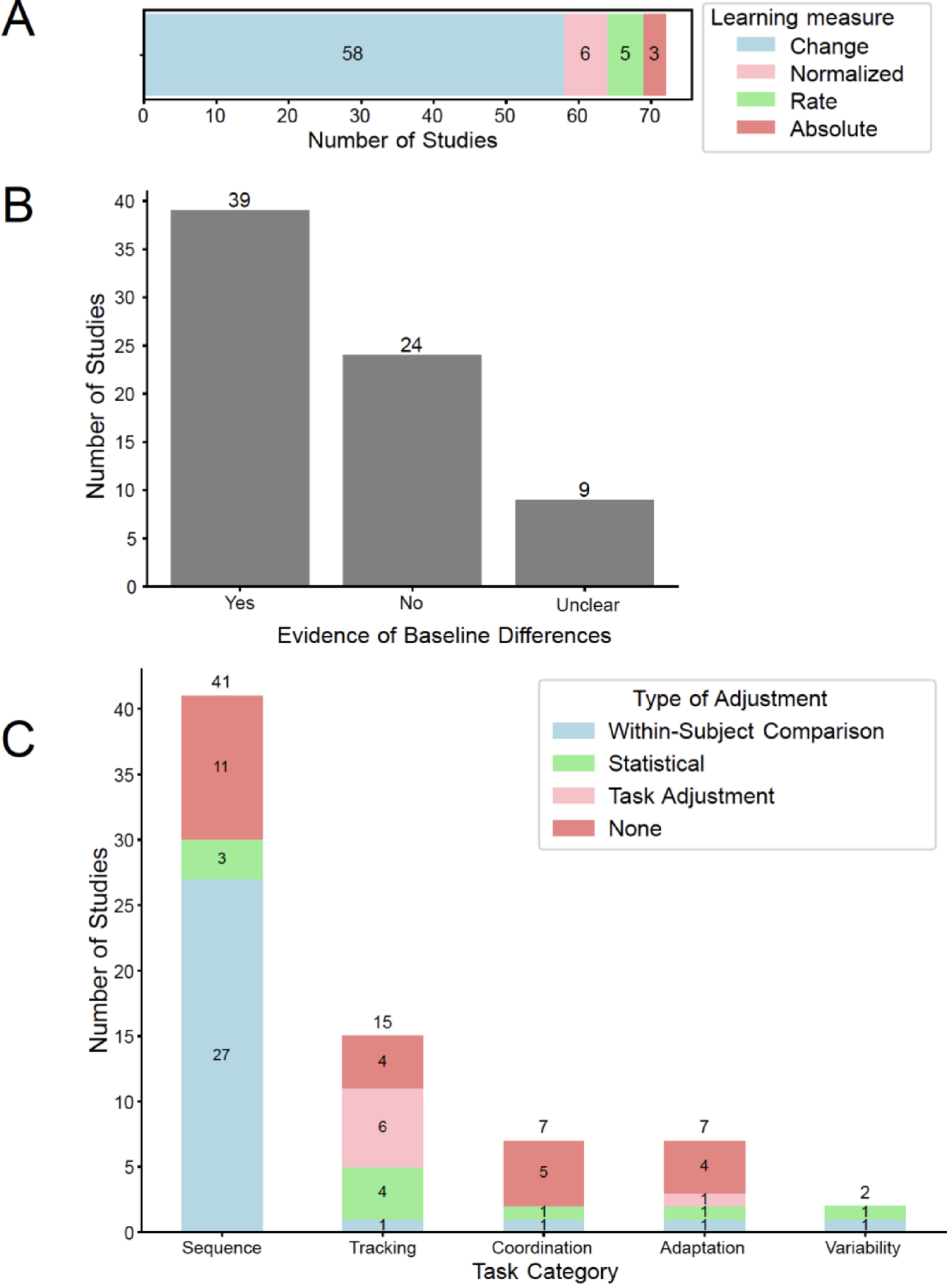
**A** Type of learning measure used for assessing deficits – change scores were the most common, which can be problematic when there are baseline differences. **B** Presence of baseline differences, i.e., difference in performance at the onset of learning between the clinical and the control groups. Over half of the studies reported differences in baseline performance. **C** Accounting for baseline differences was dependent on the task paradigm. In sequence learning tasks, the majority of paradigms used a control condition with a random sequence acting as a ‘within-subject’ comparison to offset the presence of any baseline differences. However, in other paradigms, there were statistical corrections (such as adding covariates), task adjustments, or no corrections

Because baseline differences affect different learning measures differently, we quantified the types of learning measures used. The learning measure most used was a change score (*n* = 58, 80.5%) (Fig. 4A). This change score typically involved examining performance using a Group x Block interaction where Block could represent time (e.g., early vs. late in learning) or conditions (e.g. a practiced sequence vs. a random sequence). Other measures such as normalized change (*n* = 6, 8.3%), learning rates (*n* = 5, 6.9%) and absolute levels (*n* = 3, 4.1%) were also used, albeit much less frequently.

About a little more than half of the studies (*n* = 39, 54.1%) reported significant differences in motor performance at baseline (Fig. 4B). The remainder reported no statistically different difference (*n* = 24, 33.3%) or this information was unclear (*n* = 9, 12.5%).

Strategies to account for baseline differences also tended to be dependent on the type of task being studied (Fig. 4C). Using a within-subject control was the most common way to account for baseline differences (*n* = 31, 43.1%) and was extensively used in sequence learning paradigms. Other experimental paradigms tended to adjust for baseline differences statistically (*n* = 10, 13.9%). Some tasks also adjusted task difficulty (*n* = 7, 9.7%) to ensure that baseline performances were equivalent between groups. Critically, a relatively large number of studies applied no correction for baseline differences (*n* = 24, 33.3%), even when significant baseline differences were identified (*n* = 12 out of 39, 30.8%).

### Sample sizes tended to be small and time duration of practice was short

**Fig. 5:**
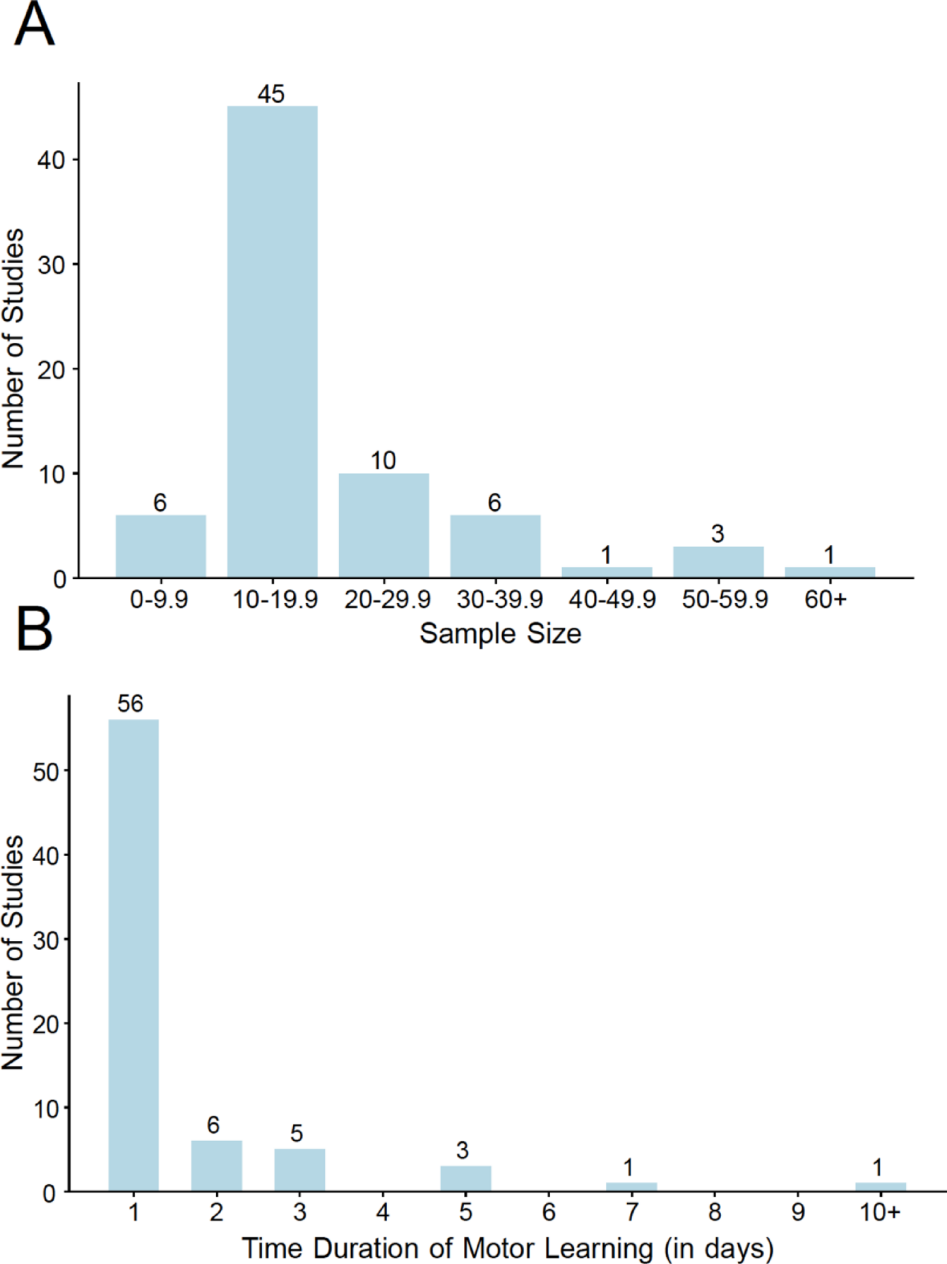
**A** Sample size distribution across studies. Sample sizes were low, but typical of motor learning studies in the literature (~ 10–20/group). **B** Time duration of motor learning across studies. A majority of the studies focused only on a single day of practice, again typical of most motor learning studies

A majority of the studies (*n* = 51, 70.8%) reviewed used an average group sample size less than or equal to 20 (Fig. 5A). In terms of time duration of practice, most studies (*n* = 56, 77.7%) were conducted within a 1-day interval, with only a handful of studies (*n* = 5, 6.9%) going beyond 3 days of practice (Fig. 5B).

## Discussion

The purpose of this review was to critically evaluate the evidence of motor learning deficits across a wide range of neurological conditions, focused on both conceptual and methodological issues. Across different motor learning paradigms, there was significant variability in the results reported as evidence for the claim of motor learning deficits including: (i) the type of deficits reported and the type of learning measures used to infer learning deficits, (ii) reporting of baseline differences and correction strategies, and (iii) the sample size and time duration of motor learning investigated. From a conceptual standpoint, we found that a large percentage of studies made claims of motor learning deficits even though about half of the studies indicated there were baseline differences in motor performance between experimental and control groups. From a methodological standpoint, we found that most studies used relatively small sample sizes with short time durations of practice. Overall, these conceptual and methodological issues highlight the significant ambiguity in the evidence for motor learning deficits in the literature.

### Conceptual issues

#### Quantifying evidence for motor learning deficits is non-trivial

A central challenge in motor learning is that there is a large degree of fragmentation in defining what constitutes learning (Ranganathan et al. 2021). The use of different measures (e.g., final performance level, change scores, relative change scores, rates of learning) to assess a learning deficit is a non-trivial issue because each type of measure assumes a different model of learning. Furthermore, prior work has shown that even when a specific learning measure like ‘rate of learning’ is used, the same measure can be operationalized in very different ways (e.g., linear slopes, single or double exponentials, power laws) (Ranganathan et al. 2022a). This fragmentation makes it challenging to reliably establish evidence of motor learning deficits and compare them across studies.

This ambiguity in interpretations of learning deficits is further exacerbated when there are baseline differences between groups. Several prior papers on motor learning (Kitago and Krakauer 2010; Schmidt 1972), have argued that in the presence of baseline differences, the same data can be used to argue that a group learned ‘more’ or ‘less’ depending on how the measure of learning is computed (as shown in Fig. 1). For example, using a change score from pre- to post-test (or equivalently, a group × time interaction) relies on an additive model of learning, where the central assumption is that that the change score is independent of the baseline performance. However, this assumption may not be justifiable in many experimental paradigms, because learning curves tend to plateau over time (i.e., participants who have high performance levels have less room to improve and, therefore, are likely to show smaller change scores). Even in cases where the underlying measure accounts for such behavior - e.g., the learning rate parameter of an exponential curve (Krebs et al. 2001), direct comparison of rates is not always straightforward since the form of these functions is quite sensitive to averaging and noise that is often seen in real-world data (Newell et al. 2001; Olivier et al. 2021) and may require additional robust fitting procedures (Zhang et al. 2025). Moreover, learning curves can take a wide range of functional forms (e.g., S-shaped curves) (Liu et al. 2006), which makes estimation and comparison of learning rates across tasks and individuals challenging.

#### Fixing the issue of baseline differences

The issue of how to account for any baseline differences is also non-trivial, especially in the current context of non-randomized studies, where individuals cannot be randomly assigned to different groups. From a statistical standpoint, Lord (Lord 1967) stated, “there simply is no logical or statistical procedure that can be counted on to make proper allowances for uncontrolled pre-existing differences between groups” (p. 305). Similarly, Kitago and Krakauer (Kitago and Krakauer 2010) refer to the same issue as “a formidable challenge” (p.1250). This has been an ongoing discussion in the statistics literature with the suggestion that while using covariates is a somewhat better strategy compared to using a change score (Senn 2006), the standard experimental design with only two time point measures (pre-test/post-test) is insufficient for an accurate estimate of the effect since it is not possible to separate ‘exogenous change’ (i.e., the change caused by learning) from ‘random change’ (Tennant et al. 2022).

Attempts to ‘equate’ baseline differences between populations by adjusting task difficulty also have challenges. Although adjusting the task to the individual is necessary in some contexts so that the task is feasible (e.g., adjusting a force production task based on the maximum voluntary isometric contraction), in the context of motor learning, adjusting task difficulty can alter the task in ways that are not easy to predict (Willingham et al. 1997). For example, adjusting task difficulty in tracking tasks can alter the shape of the learning curve (Bahrick et al. 1957). Moreover, adjusting parameters like movement speed may also alter the underlying control strategy in terms of the use of feedback and feedforward processes. These highlight the difficulty of interpreting evidence for learning deficits across populations when baseline performance is different.

Perhaps the more convincing evidence for baseline corrections comes from ‘within-subject’ comparisons – where the participant’s own performance in another condition can be used as a control condition to rule out confounds. For example, in serial reaction time tasks, the same participant is tested in the learned sequence and a random sequence, and the difference between these two conditions is assumed to be a specific measure of skill, which minimizes contamination of performance effects (Robertson 2007). However, because these involve comparisons between conditions, these also raise similar issues (i.e., do we compare the absolute change, relative change, etc.). Moreover, these types of designs cannot be easily applied to all types of task paradigms because they require the ability to measure performance in a ‘control’ condition which is a variation of the motor task that can be performed without learning.

### Methodological issues

#### Low sample size

Consistent with prior reviews on motor learning (Lohse et al. 2016; Ranganathan et al. 2021), our review indicated that sample sizes tend to be under 20. While the difficulty of recruiting clinical populations must be recognized, there are well-known issues with using small sample sizes like low power and the inflation of effect sizes (Button et al. 2013). In the current context, this lack of power is especially critical since the primary method of quantifying motor learning deficits involves detecting ordinal interactions (e.g., when both groups learn but one group learns more than the other). These interactions typically require higher power than main effects because the expected effect sizes are smaller (Lakens and Caldwell 2021). Moreover, interpreting non-significant differences in baseline performance as evidence of ‘no baseline differences’ can also be problematic in studies with low power. The latter point about inflation of effect sizes is due to a combination of suboptimal power and the use of selection thresholds (Ioannidis 2008), i.e., because small sample sizes can only detect large effects, only studies that overestimate an effect size will show statistically significant results (Button et al. 2013).

#### Short time duration of practice

Although the duration of the practice is often seen as a convenience variable (i.e., clinical assessments must ideally be short), there is a danger in using short durations of practice for assessing motor learning deficits. First, the use of short practice durations can often be problematic in clinical populations, who may require greater time to familiarize themselves with novel experimental settings or task instructions. Second, given that motor learning occurs over different time scales of practice, short-term studies primarily tap into the ‘fast time scales’ of learning which are not only transitory (Newell et al. 2009), but may also involve different learning processes related to cognition and explicit learning (McDougle et al. 2015). This overemphasis on the early phase of learning also blurs the distinction between whether participants show persistent deficits (i.e., participants do not reach the same level of performance regardless of the duration of practice) or whether they are simply slower learners (i.e., participants can reach the same level of performance but do so at a slower rate). Last, the use of short-duration experiments also reduces the relevance for tailoring rehabilitation strategies (Olson et al. 2019), which may require weeks or months of training.

#### Narrow emphasis on sequence learning paradigms

Finally, most of the work was based on the sequence learning paradigm. Although this is somewhat expected in the specific context given the theoretical backdrop of procedural/implicit learning (Penhune and Steele 2012) and the relative ease with which the paradigm can be studied even online (Bönstrup et al. 2020; Weinberger and Green 2022; Cubillos et al. 2023), the narrow focus on this one paradigm also raises the question of what aspects of motor learning are being captured. Sequence learning paradigms typically place relatively less emphasis on the actual movements themselves (instead typically focusing only on movement outcomes such as times and errors), which can obscure certain types of deficits such as the control of variability or multi-segment coordination. This lack of clinically relevant paradigms was also noted in a prior meta-analysis on implicit learning in stroke (Kal et al. 2016), which found only a single article (out of 20 included in the review) that examined learning using a whole-body coordination task. The lack of sufficient representation of other types of paradigms, coupled with the general observation that studies included in the review tended to be lab-based research (which is early in the translation pipeline), raises the risk of overgeneralization to rehabilitation based on tasks with limited ecological validity (Ranganathan et al. 2022b).

### Recommendations

In light of these challenges in evaluating evidence of motor learning deficits, we provide the following recommendations. We use our prior work on general guidelines for motor learning studies(Ranganathan et al. 2022c) as a basis for these recommendations, but specifically tailor these recommendations to address the challenges raised in the current context.

#### Characterizing participant abilities at baseline

Because motor learning in many tasks involves the integration of sensory, motor, and cognitive abilities, and baseline differences in these abilities can affect conclusions, there is a need for better characterization of participant abilities at baseline. Therefore, in addition to condition-specific assessments (such as the Unified Parkinsons’ Disease Rating Scale for Parkinson’s Disease or the Fugl-Meyer for stroke), the use of standardized test batteries such as the NIH toolbox (Gershon et al. 2013) or physiological profile assessments (Lord et al. 2003; Ingram et al. 2019) that evaluate different sensory, motor, and cognitive components across multiple neurological conditions can help provide critical information to make inferences about any observed deficits in motor learning tasks. These assessment batteries also provide estimates of heterogeneity within these populations, which can be useful for (i) assessing if impairment levels in participant sample is representative of the population (especially because the inability to perform the motor task is used as an exclusion criterion for such experiments (Willingham et al. 1997) and (ii) individual-level analyses that go beyond characterizing average differences at the group level (highlighted later in this section).

#### Building a more robust evidence base with richer experimental designs

Given the learning-performance distinction, our view is that the standard experimental approach of comparing an experimental group against a neurologically unimpaired control group in a single experimental condition is, by itself, not fully compelling evidence of a learning deficit (Ranganathan et al. 2022c). Between-subject designs provide preliminary evidence of an impaired learning mechanism, but given the potential confound of baseline differences, the exact nature of learning deficits may become more apparent when combined with ‘within-subject’ experimental designs that compare learning within the same population using different conditions or training paradigms. For example, prior work has shown that when making reaching movements, cerebellar patients have deficits adapting to a force field when the force field was introduced abruptly, but not when the same force field was introduced gradually over several trials (Criscimagna-Hemminger et al. 2010). An alternative approach that could address the issue of baseline differences is using stratified sampling based on baseline performance (Kernan et al. 1999; Ziv et al. 2020; Krishnan et al. 2025), which can be used in contexts where there is some likelihood of finding individuals in different groups with similar baseline performance (e.g., comparing two different clinical populations). In some contexts, post-hoc matching algorithms could also be used to identify how much of the observed effect in an experiment is likely due to mismatched baselines (Mandava et al. 2010).

Similarly, quantifying learning using paradigms that test participants across longer time scales could also provide more insight. For example, by measuring performance across multiple days in a throwing task in individuals with Parkinson’s disease (instead of relying on a single retention test), it was possible to distinguish motor control deficits from retention deficits (Pendt et al. 2011). These experimental designs may also provide the ability to use more sophisticated statistical models for longitudinal data such as mixed regression models (Lohse et al. 2020) that incorporate time more explicitly into the model.

#### Developing model tasks and pre-registration

In light of difficulties in identifying appropriate models and variables across various paradigms, a proposed solution for cumulative science is to develop model task paradigms that can be compared across different studies (Ranganathan et al. 2021). In the context of motor learning deficits, there are three specific challenges that need to be addressed. First, the goal is to develop tasks where deficits are not ‘masked’ by motor impairments (Subramanian et al. 2018) or the use of alternative strategies to perform the task (Nicolson and Fawcett 1994). For example, in several task paradigms, there are both explicit and implicit processes involved in learning (Taylor and Ivry 2013), and require specific experimental manipulations to distinguish the relative contribution of these processes. Second, developing tasks that are sensitive enough to detect group differences and associated with specific underlying learning mechanisms must be balanced with relevance to real-world contexts such as rehabilitation. This means that task paradigms need to: (i) focus on movement quality and coordination in addition to task outcome variables such as times and errors, and (ii) be relatively complex enough so that longer timescales of learning beyond a single session can also be examined (i.e., so that performance does not reach an asymptote within a single session). Given the wide discrepancy between basic research (which typically deals with a single learning mechanism) and real-world contexts (which typically involve multiple learning mechanisms operating at different time scales), bridging this gap between basic research and clinical practice requires clear delineation of different stages of investigation, choosing tasks that are optimal for each stage, and triangulating the evidence across these stages. Third, pre-registration could help address the issue of fragmentation in how learning is quantified. Specifying and justifying in advance the type and calculation of the learning measure, sample size justification, outlier exclusion criteria, and accounting for baseline differences could provide more transparency into the choices of these methods. Finally, in cases where issues such as baseline differences exist, examining the sensitivity of the conclusions to the type of learning measures or the method of accounting for baseline differences would increase the robustness of the research.

#### Greater focus on individual-level data

In addition to theoretical insight, one goal of measuring motor learning deficits in neurological populations is that it can potentially serve as a non-invasive behavioral marker for neurological function and be used as an early predictor of disease states such as Alzheimer’s disease (Ruitenberg et al. 2023; Korte et al. 2024), Parkinsons’ disease (Gigi et al. 2024), Huntington’s disease (Ghilardi et al. 2008) and spinocerebellar ataxia (Bando et al. 2019). However, most of the studies reviewed tended to focus on group-level differences (e.g., by showing statistically significant differences using ANOVAs or t-tests) which can be misleading given the heterogeneity in many neurological conditions. In addition to more comprehensive baseline assessments, increasing transparency by including individual data in figures (Weissgerber et al. 2015) and incorporating appropriate statistical reporting for biomarkers (e.g., by using measures that are sensitive to base rates) (Ray et al. 2010) can provide information about whether a deficit can indeed be considered predictive of future disease states and be compared with existing alternatives. The emphasis on explaining individual data, coupled with the choice of specific model tasks, could also bolster computational models of learning (Scheidt et al. 2001; Izawa and Shadmehr 2011; Schweighofer et al. 2011; Schlerf et al. 2013; Therrien et al. 2016; Chu et al. 2016; Filoteo et al. 2017) that can provide more insight into ‘why’ performance of two groups are different, rather than simply stating ‘that’ they performed differently.

There are a couple of important limitations associated with the study. First, because we did not adopt an explicit definition of motor learning, the review included papers that do not have a common definition of motor learning. Although one advantage of casting a broad net is that it allowed us to capture and document the range of learning paradigms commonly used to assess deficits in clinical populations, a downside of including multiple learning paradigms is that it somewhat limited the specificity of the recommendations. As mentioned earlier, motor learning is a complex process spanning multiple phenomena (Krakauer et al. 2019; Tsay et al. 2024) over multiple timescales (Newell et al. 2001) and it is our view that no single definition of learning applies universally across all paradigms. For example, one definition of motor learning emphasizes the use of delayed retention tests (e.g., measuring performance after a 24 h delay) to assess relatively permanent changes in behavior (Schmidt and Lee 2005). However, it is important to note that while a single retention test can account for performance effects that dissipate over time (such as fatigue), it does not account for performance effects due to impairment or motor control deficits. Therefore, rather than using a ‘one-size-fits-all’ solution for all learning studies, future work that adapts our recommendations to address specific learning mechanisms and task contexts is critical. Second, from the viewpoint of translational impact, it is important to note that motor learning is only one component of neurorehabilitation, and that interventions have to be considered in the context of interaction between different levels of analysis of the ICF framework (body, function, activities, and participation) (Stucki et al. 2002). In other words, neurorehabilitation interventions can have different objectives such as reducing impairment, addressing activity limitations, or removing an environmental barrier (Lexell and Brogårdh 2015). Therefore, the presence of motor learning deficits in a clinical population should not be taken as directly indicative of the effectiveness of neurorehabilitation per se.

In summary, our review highlights that addressing motor learning deficits in the neurological population is a challenging research question. A central contribution of our paper is to document how variations in seemingly trivial choices in terms of researcher degrees of freedom (baseline correction, task selection, time duration of learning) across a range of motor learning paradigms result in ambiguous interpretations, which potentially explain why there is no consensus view on these issues despite several decades of work. We want to emphasize that there are no ‘quick fixes’ to the issues raised here; instead, the answer to this challenge requires the development of new research approaches with more representative tasks and new experimental designs. A more rigorous, convergent approach using multiple lines of evidence will eventually be necessary to create theoretical and clinical impact.

## Data Availability

No datasets were generated or analysed during the current study.
